# Developmental arrest rate of an embryo cohort correlates with advancing reproductive age, but not with the aneuploidy rate of the resulting blastocysts in good prognosis patients: a study of 25,974 embryos

**DOI:** 10.18632/aging.206328

**Published:** 2025-10-10

**Authors:** Andres Reig, Emre Seli

**Affiliations:** 1 IVIRMA Global Research Alliance, IVIRMA New Jersey, Basking Ridge, NJ 07920, USA; 2 Department of Obstetrics, Gynecology and Reproductive Sciences, Rutgers University, Robert Wood Johnson Medical School, New Brunswick, NJ 08901, USA; 3 Department of Obstetrics, Gynecology and Reproductive Sciences, Yale School of Medicine, New Haven, CT 06510, USA

**Keywords:** ovarian aging, reproductive aging, embryonic arrest, embryonic aneuploidy, developmental arrest

## Abstract

This study aimed to investigate the extent to which developmental arrest rate in embryos generated using assisted reproduction correlate with female age and the rate of aneuploidy in the cohort. A total of 25,974 embryos from 1,928 cohorts were included in the study, with an overall embryo developmental arrest (EDA) rate of 40.3% (95% CI: 39.8–40.9%). The median EDA rate increased with age: 33.0% (IQR: 22.0–50–0%) in <35 years old, 38.0% (25.0–50.0%) in 35–37 years old, 40.0% (29.0–54.0%) in 38–40 years old, 44.0% (38.8–56.5%) in 41–42 years old, and 44.0% (40.0–58.0%) in >42 years old; *p* < 0.0001. A very weak positive correlation was identified between EDA rate and the rate of aneuploidy (r: 0.07, 95% CI 0.03–0.11; R^2^: 0.00, *p* < 0.01) when evaluating all cohorts. However, when adjusting for age, no statistically significant relationship between aneuploidy and EDA was observed. Our findings suggest that the rate of EDA and the rate of whole chromosome aneuploidy in the resulting blastocyst cohort are both associated with female age, but not with each other. Therefore, EDA and aneuploidy rates represent two independent factors in determining the number of euploid embryos available for transfer and the overall likelihood of ART success.

## INTRODUCTION

The success of assisted reproduction through *in vitro* fertilization (IVF) is largely determined by the cumulative pregnancy rate (CPR), defined as the rate at which patients who start a treatment cycle ultimately achieve pregnancy. As has been shown in recent years, subsequent embryo transfers after a prior failed one carry similar pregnancy rates [1], showcasing that recurrent implantation failure is rare in patients undergoing euploid embryo transfers with an anatomically normal uterus. Therefore, the chief predictor of IVF success is the availability of embryos with reproductive potential. This, in turn, is determined by three step-wise factors: the size of the mature oocyte cohort retrieved (a function of ovarian reserve and ovarian response to stimulation), the blastocyst formation rate among those oocytes (requiring both fertilization and subsequent embryonic development through the blastocyst stage), and the euploidy rate among the available blastocysts (whether this is known by way of genetic testing or unknown).

To fully understand the impact of embryo developmental arrest (EDA) on IVF outcomes, it is important to study its contribution independently, as well as how these three factors affect one another. This is particularly crucial because these phenomena not only occur consecutively in the timeline of an IVF cycle but also collectively contribute to the underlying effect of female age on most, if not all, processes affecting reproductive efficiency. Indeed, increasing female age has been clearly correlated with increased embryonic aneuploidy [2] and with decreased ovarian reserve and likelihood of obtaining a euploid embryo [3, 4]. Two recent studies have also shed light on the relationship between age and blastocyst formation, revealing decreased blastulation rates when comparing women 41 years old and older with those under 41 [5, 6]. Another study evaluating the relationship between maternal age and specific morphokinetic markers of embryo development concluded that increasing maternal age is associated with key functions of embryonic development separate from meiotic aneuploidy [5].

The specific mechanisms involved in the developmental arrest of embryos prior to reaching the blastocyst stage are the focus of several recent and ongoing studies (reviewed in [7]). Genes that encode factors that are present in the oocyte and required for early embryonic development are called maternal effect genes [7]. Mutations in maternal effect genes, such as *TUBB8,* which is involved in spindle assembly, have been linked with developmental arrest of early through cleavage stage embryos [8, 9]. Impaired mitochondrial function, long the target of embryonic competence research, can also result in an inability to progress to blastocyst. Animal models in which the pathway for breaking down mitochondrial unfolded proteins was impaired showed no progression to blastocyst and as well as low numbers of mature oocytes [10, 11]. Similarly, targeted deletion of *MFN2*, which mediates mitochondrial fusion, results in lower blastocyst formation [12].

In the current study, we hypothesized that while EDA and aneuploidy are both affected by maternal aging, these two processes are likely independent from each other and a higher embryonic arrest rate does not correlate with a higher aneuploidy rate in the resulting blastocyst cohort. Should this prove to be the case, the research being performed in understanding said arrest would provide an avenue not just for understanding embryonic biology, but for developing potential therapeutic targets to further the treatment of infertility.

## RESULTS

1,928 embryo cohorts were included in the study. These included 25,974 fertilized oocytes (2PN), resulting in 15,495 biopsied blastocysts, representing an overall EDA rate of 40.3% (95% CI: 39.8–40.9%).

### Developmental arrest rate increases with age

**Figure 1:**
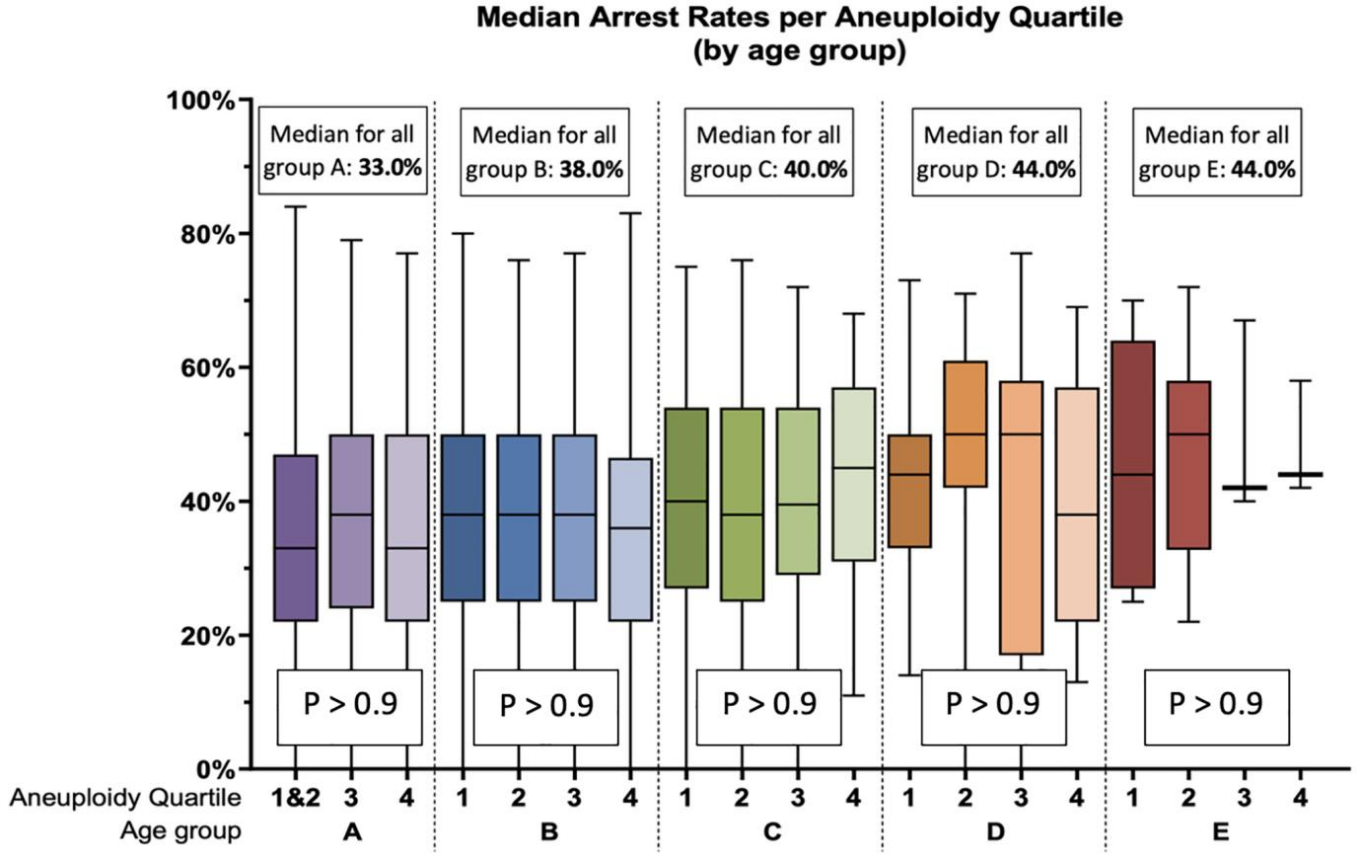
Arrest rate, by SART age group (**A**–**E**) and by aneuploidy rate quartile. The median arrest rates for all cycles in each age group, regardless of aneuploidy rate, are shown in the white boxes. Aneuploidy rate quartiles (1–4) were calculated separately for each age group. The horizontal line represents the median, the box represents the interquartile range, and the whiskers represent the total range. No statistically significant differences were found within each age group.

The median EDA rate per cohort in each age group was calculated and compared. The results of this analysis demonstrate a statistically significant increase in the median rate of arrest with increasing age: 33.0% (IQR: 22.0–50–0%) in <35 years old (group A), 38.0% (25.0–50.0%) in 35–37 years old (group B), 40.0% (29.0–54.0%) in 38–40 years old (group C), 44.0% (38.8–56.5%) in 41–42 years old (group D), and 44.0% (40.0–58.0%) in >42 years old (group E), *p* < 0.0001 (Figure 1).

Post-hoc comparisons between individual groups revealed that the significant differences are specifically between group A and groups C, D, or E, but no statistical significance between any age group and the next, revealing a gradual worsening in arrest rate that is only apparent when enough time has elapsed.

### Relationship between developmental arrest and aneuploidy

**Figure 2:**
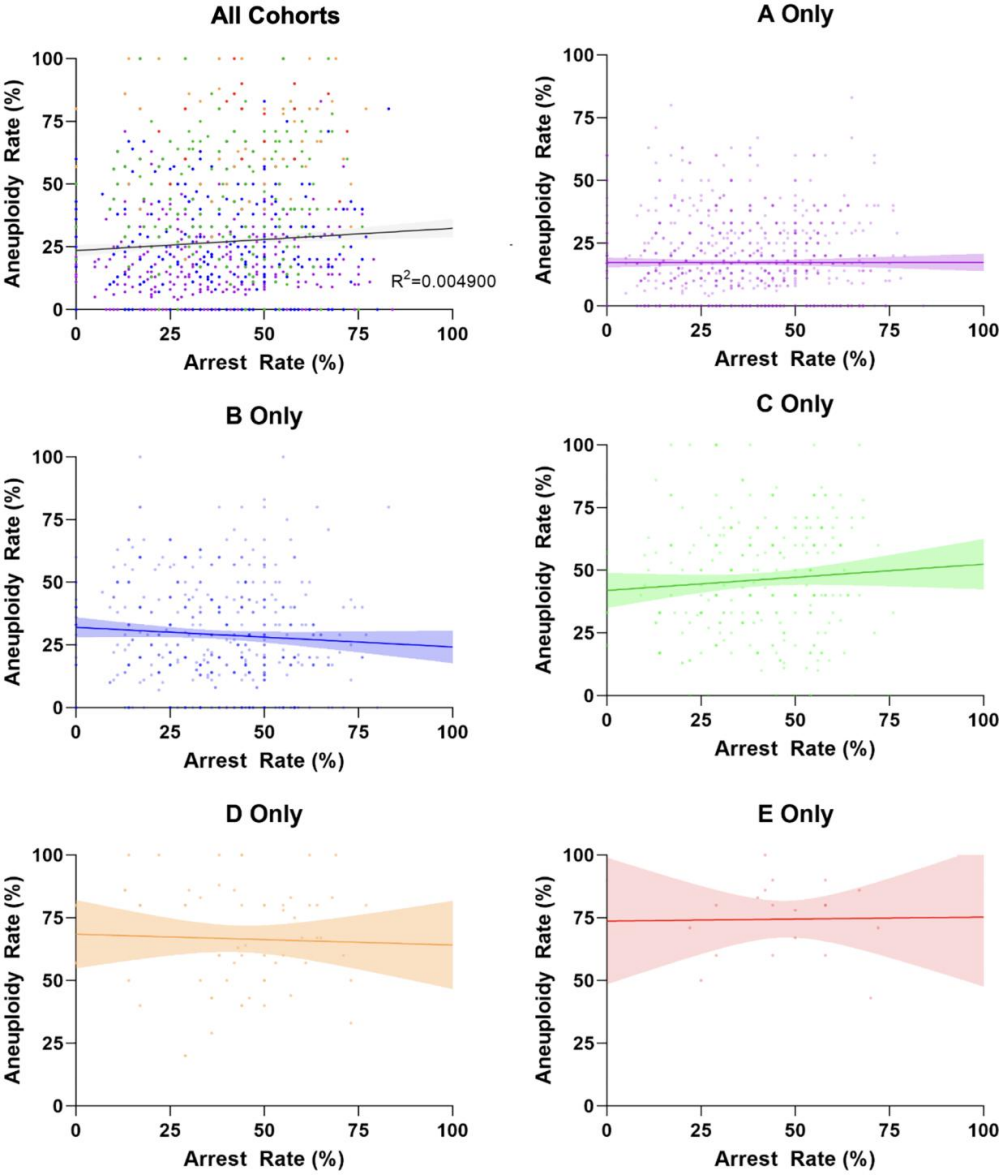
**Correlation between arrest rate and aneuploidy rate per age group.** Each plotted data point is an individual embryo cohort. The color of each individual data point matches the age groups in Figure 1. The lines represent the linear regression best fit (R^2^ for Pearson correlation and best fit line with 95% confidence interval), with all R^2^ values being less than 0.01 and revealing no correlation between both parameters.

Before accounting for the effect of age, the correlation between EDA rate and whole chromosome aneuploidy rate was evaluated. All 1,928 embryo cohorts included in the study are plotted in Figure 2 according to their individual arrest and aneuploidy rates. A very weak – yet statistically significant – positive correlation was identified (r: 0.07, 95% CI 0.03–0.11; R^2^: 0.00, *p* < 0.01).

### Higher rate of aneuploidy in an embryo cohort is not associated with an increased arrest rate

To account for the effect of age, the relationship between arrest and aneuploidy was further analyzed by two separate approaches. First, the cohorts in each age group were divided into aneuploidy quartiles, calculated separately for each age group (Table 1). Of note, as patients under 35 years of age have very low rates of embryo aneuploidy, more than 25% of patients in group A had an aneuploidy rate of 0%, making it impossible to determine a threshold between the first and second quartile, therefore resulting in the first and second quartiles being grouped together. All other age groups were split into 4 quartiles.

**Table 1:** Breakdown of aneuploidy rate thresholds for determining quartiles within each age group, along with average arrest rates for each quartile.

**Age group**	**Aneuploidy quartile range**	**Arrest rate**
A	0.0–0.0%	N/A
	0.0–16.7%	47.3% (4717/5251)
	16.7–25.0%	47.9% (1558/1696)
	25.0–83.3%	48.9% (2005/2098)
**Total A**	**0.0–83.3%**	**47.8% (8280/9045)**
B	0.0–16.7%	50.0% (1252/1254)
	16.7–25.0%	50.5% (794/778)
	25.0–40.0%	49.9% (957/961)
	40.0–100.0%	48.1% (878/948)
**Total B**	**0.0–100.0%**	**49.6% (3881/3941)**
C	0.0–29.6%	52.1% (553/508)
	29.6–44.4%	49.1% (516/534)
	44.4–60.0%	50.6% (512/499)
	60.0–100.0%	54.1% (529/448)
**Total C**	**0.0–100.0%**	**51.5% (2110/1989)**
D	20.0–50.0%	57.7% (158/116)
	50.0–66.7%	60.1% (152/101)
	66.7–80.0%	56.9% (115/87)
	80.0–100.0%	53.1% (104/92)
**Total D**	**20.0–100.0%**	**57.2% (529/396)**
E	42.9–60.0%	58.2% (39/28)
	60.0–80.0%	63.2% (84/49)
	80.0–85.7%	61.5% (32/20)
	85.7–100.0%	52.6% (30/27)
**Total E**	**42.9–100.0%**	**59.9% (185/124)**

The arrest rate was then analyzed between aneuploidy quartiles within each age group. As shown in Figure 1, no statistically significant differences were found in arrest rates with increasing aneuploidy rates. Moreover, within each age group, the median arrest rate was not found to consistently increase from one aneuploidy quartile to the next (Figure 1).

In the second approach, the Pearson correlation between arrest rate and aneuploidy rate was evaluated separately for the cohorts in each age group. All R^2^ values were under 0.01, representing no correlation between arrest and aneuploidy rates in any of the age groups (Figure 2).

## DISCUSSION

To our knowledge, this is the first study to evaluate the relationship between EDA and aneuploidy of the resulting blastocyst cohort in the context of female age. We evaluated the relationship between the EDA rate in a cohort of embryos and the rate of whole chromosome aneuploidy in the resulting blastocysts, taking female age into account as the main factor affecting both measures. The rate of EDA was found to increase with age: from a median arrest rate of 33% in the youngest group to 44% in the oldest. The very weak correlation between EDA and aneuploidy disappeared when evaluating this within each age group and the rate of arrest was seen not to increase through increasing aneuploidy rate quartiles at the same ages, thus revealing that these two parameters are associated through their common denominator of age, but appear to be independent of each other.

This study has some limitations, including its retrospective nature. However, this allowed for the evaluation of a very large dataset of almost 2,000 cohorts, over 25,000 fertilized oocytes, and more than 15,000 biopsied embryos. Furthermore, this large sample size yielded enough cohorts to stratify by aneuploidy quartiles with high granularity (down to 20% aneuploidy rate intervals given inclusion only of cohorts with at least 5 blastocysts biopsied), even after adjusting by age. This decision – to exclude cohorts in which <5 blastocysts were available for PGT-A – inherently biases the study population to an extremely good prognosis one, particularly in the groups of more advanced female age. However, allowing cohorts with only 1 blastocyst, for example, could have resulted in an aneuploidy rate of 0%, which would have had the same weight in the analysis as a 0% rate in a cohort with 8 blastocysts. Therefore, while this results in an unusually excellent prognosis patient group, we believe that the findings made from a biological perspective as pertains to the relationship of aneuploidy and arrest remain valid for smaller cohorts.

Regarding PGT-A results, it should be noted that we evaluated the correlation between EDA and whole chromosome aneuploidy, thus purposely not including in this definition embryos which could potentially have whole chromosome mosaicism, segmental mosaicism, or other abnormalities. At our centers, we do not receive reporting for secondary PGT-A findings unless specifically requested by each individual physician, following previously published evidence that these could continue to have reproductive potential [13, 16]. These other forms of aneuploidy should be further correlated with EDA. Furthermore, it is important to note that the results of this study should not be interpreted as supporting nor discouraging from the use of PGT-A, as it did not evaluate the usefulness of PGT-A in anyway. Rather, PGT-A was merely used as a test to identify the correlation between arrest rate and aneuploidy of the remaining blastocysts.

Many of the proposed mechanisms for embryonic developmental arrest, such as mitochondrial dysfunction, impaired mitophagy, or mutations in some maternal effect genes may result in effects specific to embryo development to a blastocyst. However, others such as *TUBB8* or *TRIP13* mutations, could affect both embryonic development and euploidy, as they affect the process of cell division and specifically the assembly of the spindle and its checkpoints. Importantly, these maternal effect genes likely play a role before zygotic gene activation, which takes place at the 4–8 cell stage in human embryos, thus resulting in very early developmental arrest [7].

Several other studies have begun answering the question of whether aneuploid embryos have higher rates of arrest before arriving at the blastocyst stage, focusing on specific stages of EDA and types of associated aneuploidy. In a recent study using polar body biopsy to evaluate the ploidy status of oocytes, monosomies affected cleavage stage embryo quality more than trisomies, and more aneuploid embryos arrested than euploid ones [17]. In another study correlating the results of PGT-A with EDA, 94% of arrested embryos were found to be aneuploid (vs 69% of embryos that reached the blastocyst stage), with the vast majority of said aneuploidies being of mitotic origin. In fact, mitotic whole chromosome aneuploidy and/or segmental aneuploidy affected 72% of arrested embryos, compared to only 32% of those that developed into blastocysts, suggesting that different mechanisms of aneuploidy may be affecting embryos that arrest vs those that develop into blastocysts [18].

Our study did not seek to evaluate whether aneuploidy results in embryonic developmental arrest. Indeed, answering said question would have required testing arrested embryos for aneuploidy, which poses significant challenges ranging from logistical issues to methodological ones. Any study seeking to evaluate the rate of aneuploidy in arrested embryos must, by definition, first make the decision that an embryo’s development has arrested. In order to do this, particularly in the setting of real patients and not experimental embryo cohorts, there must be a wait period after which the embryo is deemed not to develop any further. Depending on the clinical setting and the method of practice of each individual center, some will continue to keep these embryos in culture for all 5–7 days of culture despite their likelihood of being arrested, only discarding them (or in this case analyzing them for aneuploidy) after this extended culture. This poses some challenges, as the DNA within a cell of a potentially euploid embryo that arrested two days before being analyzed may have degraded unevenly, resulting in a potentially false non-euploid result. Furthermore, particularly in the context of embryo mosaicism, it is unknown whether cells (and their DNA) that are aneuploid degrade faster or slower, which would result in the interpretation of any analysis being dependent on when it was conducted (how many days from the actual arrest).

Instead, our study sought only to identify a potential correlation between the arrest rate in an embryo cohort and the aneuploidy rate in the resulting blastocysts only, excluding those arrested (not the entire cohort of embryos). From a clinical standpoint, we believe this knowledge can be useful for patient counseling, particularly while awaiting PGT-A results. From a biological point of view, while our study was not intended to elucidate a causal relationship between aneuploidy and embryonic arrest, the lack of correlation does suggest that there are different mechanisms at play in embryos that arrest (euploid or aneuploid) and those that are aneuploid but otherwise progress normally to the blastocyst stage.

While this study has evaluated embryo developmental arrest as a whole, including as such any fertilized embryos that did not become a useable blastocyst, different developmental mechanisms may be involved in embryonic arrest at different stages. As such, future studies evaluating embryos that arrest at different stages, as well as the relationship of these stage-specific arrests with aneuploidy, would add to our understanding of this phenomenon. The genetic and metabolic analysis of arrested embryos, while challenging for the reasons mentioned above, will prove of paramount importance to addressing embryo developmental arrest.

This is a promising time for our field, in which future research will continue to evaluate arrested embryos at every stage for their ploidy status, perhaps through single-cell genetic analysis to truly analyze the mitotic versus meiotic nature of the observed aneuploidies, thus shedding light onto the question of whether aneuploid embryos arrest more frequently. More importantly, the isolation of developmental arrest from meiotic aneuploidy has the potential to allow for the identification of novel therapeutic targets in ART – such as mitochondrial function or even gene therapy for specific mutations resulting in embryonic arrest – which may increase the number of euploid embryos available for transfer in a given cycle.

## CONCLUSION

The rate of EDA and the rate of whole chromosome aneuploidy in the resulting blastocyst cohort are both associated with female age, but not with each other. While aneuploidy itself – and some of the mechanisms leading to it – may lead to EDA and some of the mechanisms leading to EDA may result in aneuploidy, these two phenomena represent two independent factors in determining the number of euploid embryos available for transfer and the overall likelihood of ART success.

## METHODS

### Study design

This was a retrospective cohort study conducted at a single, university-affiliated, fertility center (IVIRMA New Jersey, Basking Ridge, NJ, USA). Institutional review board approval was obtained (Advarra protocol #PRO00027158).

Embryos that were derived from one cycle of egg retrieval were collectively deemed an embryo cohort. Embryo cohorts resulting from IVF cycles performed between January 2020 and December 2021 were included, and only the first cycle for each patient was included in the dataset, with the following exclusion criteria: any cohorts in which only some of the available embryos underwent trophectoderm biopsy and PGT-A (whether for patient preference or for other reasons, such as economic), cohorts with trophectoderm biopsy for other reasons, such as preimplantation genetic testing for monogenic conditions (PGT-M) or segmental rearrangements (PGT-SR), cycles for oocyte cryopreservation, cycles in which surgically obtained sperm was used, cycles using oocytes resulting from donation.

Additionally, we made the decision to only include cohorts in which at least 5 blastocysts were available for biopsy. This decision had the aim of obtaining enough granularity in the identification of aneuploidy rate, as cohorts comprised of only 2 embryos, for example, can only have aneuploidy rates of 0%, 50%, or 100%, thus not serving the purpose of identifying the true relationship between developmental arrest rate and aneuploidy rate.

### Cycle characteristics

All patients underwent controlled ovarian stimulation with standard regimens as described previously [13, 14]. The cycles were comprised of a vast majority having stimulation by an antagonist protocol, with some cycles involving microflare or down-regulation protocols. Final oocyte maturation was triggered by injection of leuprolide acetate and/or human chorionic gonadotropin. Vaginal oocyte retrieval took place 36 hours after trigger. All meiosis II oocytes were fertilized by intracytoplasmic sperm injection (ICSI) and all resulting 2PNs were cultured to the blastocyst stage for a maximum of 7 days.

### PGT-A

Embryos underwent a trophectoderm biopsy once they reached the blastocyst stage if they were deemed usable by morphology grading. Embryos were deemed useable once they reached the blastocyst stage, and thus biopsied, if a grade of D was not observed in neither the inner cell mass nor the trophectoderm by modified Gardner’s grading system [15]. All trophectoderm biopsy samples were analyzed for whole chromosome aneuploidy by a previously validated, next-generation sequencing platform at a single genetics laboratory (JUNO Genetics, Basking Ridge, NJ, USA) as described previously [13].

### Outcome measures

All the included cohorts were divided into the Society for Assisted Reproductive Technology (SART) age groups according to the patient age at the time of COS initiation (group A: <35 years old, B: 35–37 years old, C: 38–40 years old, D: 41–42 years old, and E: >42 years old).

The primary outcome was the correlation between developmental arrest rate and the aneuploidy rate of the embryo cohorts within each age group. The developmental arrest rate was defined as the percentage of fertilized oocytes with 2 pronuclei (2PN) that did not become a useable blastocyst after culture for up to 7 days. At our center, all 2PN zygotes are cultured for five to seven days, regardless of development before day five. Blastocysts are cryopreserved on day five if they reach BB grading or better, otherwise being allowed to progress to day 6. Those that do not reach a grading of CC or better by day 6 are cultured for one additional day. Blastocysts that reach CC or better by day seven are cryopreserved, with the remainder deemed unusable. The aneuploidy rate was defined as the proportion of useable blastocysts with a whole chromosome aneuploidy.

Secondary outcomes included a comparison of the embryonic arrest rate between whole age groups and between aneuploidy rate quartiles within each age group.

### Statistical analyses

The Pearson correlation coefficient between aneuploidy and arrest rates was calculated for each age group and for all cohorts combined. R^2^ thresholds of <0.1, 0.1–0.3, 0.3–0.5, and >0.5 were used to define no correlation, low degree of correlation, moderate degree, and high degree, respectively.

For the comparison of embryonic arrest rates between age groups, Kruskal-Wallis test was utilized, and a *p*-value of 0.05 was used as a threshold for statistical significance. The same was applied for the comparison of embryonic arrest rates between aneuploidy rate quartiles within each age group.

Normally distributed variables are given as mean ± standard deviation (SD); non-normally distributed variables are given as median (interquartile range (IQR)).
